# 4-Amino-3-bromo­benzoic acid

**DOI:** 10.1107/S1600536809006825

**Published:** 2009-02-28

**Authors:** Muhammad Nadeem Arshad, M. Nawaz Tahir, Islam Ullah Khan, Muhammad Shafiq, Abdul Waheed

**Affiliations:** a Department of Chemistry, Government College University, Lahore, Pakistan; b Department of Physics, University of Sargodha, Sargodha, Pakistan

## Abstract

The asymmetric unit of the title compound, C_7_H_6_BrNO_2_, consists of two mol­ecules having a small variation of bond lengths and angles. The title compound forms dimers through pairs of O—H⋯O hydrogen bonds involving the carboxyl­ate groups. The dimers are linked into polymeric forms through inter­molecular hydrogen bonds, forming *R*_2_^1^(6), *R*_3_^2^(8) and *R*_3_^3^(15) ring motifs.

## Related literature

The title compound has been prepared as an inter­mediate for the synthesis of sulfonamides (Arshad *et al.*, 2009 ▶) and benzothia­zines (Arshad *et al.*, 2008 ▶). For hydrogen-bond motifs, see: Bernstein *et al.* (1995 ▶). For related structures, see: Pant (1965 ▶); Tanaka *et al.* (1967 ▶). For the synthesis, see: Krishna Mohan *et al.* (2004 ▶).
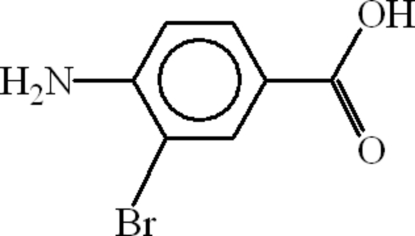


## Experimental

### 

#### Crystal data

C_7_H_6_BrNO_2_
*M* *_r_* = 216.04
Orthorhombic, 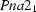

*a* = 24.3968 (11) Å
*b* = 4.8388 (2) Å
*c* = 12.8040 (5) Å
*V* = 1511.53 (11) Å^3^
*Z* = 8
Mo *K*α radiation
μ = 5.38 mm^−1^
*T* = 296 K
0.22 × 0.16 × 0.14 mm

#### Data collection

Bruker Kappa APEXII CCD diffractometer
Absorption correction: multi-scan (*SADABS*; Bruker, 2005 ▶) *T*_min_ = 0.375, *T*_max_ = 0.469
9922 measured reflections
3908 independent reflections
3169 reflections with *I* > 2σ(*I*)
*R*_int_ = 0.027

#### Refinement

*R*[*F* ^2^ > 2σ(*F* ^2^)] = 0.030
*wR*(*F* ^2^) = 0.059
*S* = 1.00
3908 reflections
214 parameters
1 restraint
H atoms treated by a mixture of independent and constrained refinement
Δρ_max_ = 0.44 e Å^−3^
Δρ_min_ = −0.49 e Å^−3^
Absolute structure: Flack (1983 ▶), 1857 Friedel pairs
Flack parameter: 0.012 (9)

### 

Data collection: *APEX2* (Bruker, 2007 ▶); cell refinement: *SAINT* (Bruker, 2007 ▶); data reduction: *SAINT*; program(s) used to solve structure: *SHELXS97* (Sheldrick, 2008 ▶); program(s) used to refine structure: *SHELXL97* (Sheldrick, 2008 ▶); molecular graphics: *ORTEP-3 for Windows* (Farrugia, 1997 ▶) and *PLATON* (Spek, 2009 ▶); software used to prepare material for publication: *WinGX* (Farrugia, 1999 ▶) and *PLATON*.

## Supplementary Material

Crystal structure: contains datablocks global, I. DOI: 10.1107/S1600536809006825/bq2123sup1.cif

Structure factors: contains datablocks I. DOI: 10.1107/S1600536809006825/bq2123Isup2.hkl

Additional supplementary materials:  crystallographic information; 3D view; checkCIF report

## Figures and Tables

**Table 1:** Hydrogen-bond geometry (Å, °)

*D*—H⋯*A*	*D*—H	H⋯*A*	*D*⋯*A*	*D*—H⋯*A*
O1—H1⋯O4^i^	0.82	1.76	2.564 (3)	165
N1—H1*A*⋯Br1	0.92 (4)	2.63 (4)	3.081 (4)	111 (3)
N1—H1*B*⋯O2^ii^	0.83 (5)	2.57 (5)	3.313 (4)	149 (4)
N2—H2*A*⋯Br2	0.99 (4)	2.68 (4)	3.099 (4)	106 (2)
N2—H2*A*⋯Br1^ii^	0.99 (4)	2.69 (4)	3.630 (4)	158 (3)
N2—H2*B*⋯O1^iii^	0.79 (5)	2.43 (5)	3.216 (4)	179 (6)
O3—H3⋯O2^iv^	0.82	1.90	2.723 (3)	178
C5—H5⋯O2^ii^	0.93	2.59	3.407 (4)	147
C12—H12⋯O4^v^	0.93	2.54	3.470 (4)	174

Symmetry codes: (i) 
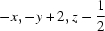
; (ii) 
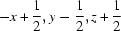
; (iii) 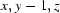; (iv) 
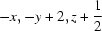
; (v) 
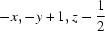
.

**Fig. 1.**
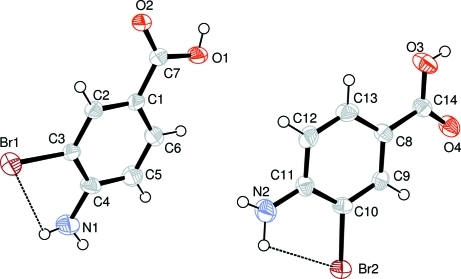
ORTEP drawing of the title compound, with the atom numbering scheme. The thermal ellipsoids are drawn at the 30% probability level. The dotted lines show the intramolecular H-bonds.

**Fig. 2.**
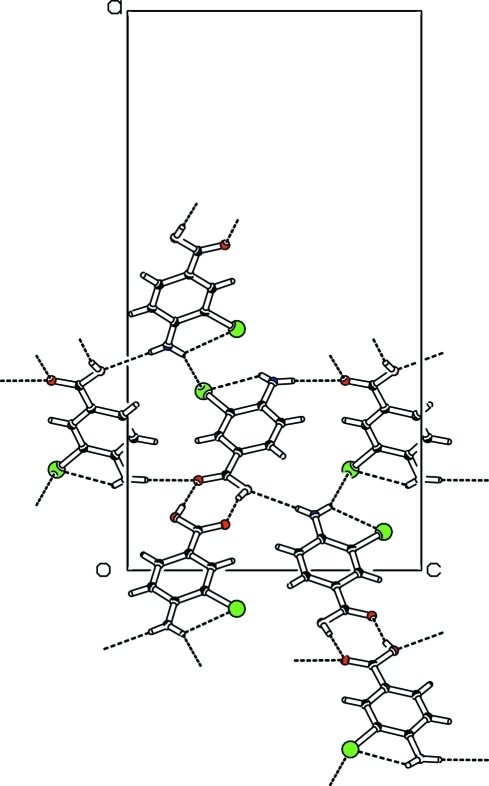
The projectional view (PLATON: Spek, 2009) which shows that molecules are dimerized and form ring motifs.

## supplementary crystallographic information

### Comment

Different types of aromatic anilines have been used for the synthesis of carboxamides and sulfonamides. The title compound (I), (Fig 1), has been prepared as an intermediate for the synthesis of sulfonamides (Arshad *et al.*, 2009), benzothiazines (Arshad *et al.*, 2008) and different metal complexes.

The crystal structure of *m*-Bromobenzoic acid (Tanaka *et al.*, 1967) and 3,5-Dibromo-*p*-aminobenzoic acid (Pant, 1965) has been published. The title compound consists of an asymmetric unit having two chemical isomers. There is a small variation of bond lengths and bond angles among the two isomers and both isomers form five membered ring (Br/C/C/N/H) through intramolecular H-bond of type N—H···Br. The molecules are dimerized forming *R*_2_^2^(8) ring motifs (Bernstein *et al.*, 1995). These dimers are linked to each other through *R*_2_^1^(6), *R*_3_^2^(8) and *R*_3_^3^(15) ring motifs (Table 1), (Fig 2).

### Experimental

The title copound was prepared following the same method (Krishna Mohan *et al.*, 2004) available in literature. 4-Amino Benzoic acid (2 g, 0.0146 mol) and ammonium bromide (1.5 g, 0.16 mol) was charged to a flask (25 ml) containing acetic acid (15 ml). Hydrogen peroxide (0.545 g, 0.016 mol) was added drop wise to the above mixture and allowed to stirr at room temperature for 3 h. Stirring was stoped and allowed it to setteled down. Precipitate obtained was filtered and washed with water and recrystalizesd in dichloromethane and methanol for X-ray studies.

### Refinement

The coordinates of H-atoms of amino groups were refined. H-atoms were positioned geometrically, with O-H = 0.82 Å for OH, C-H = 0.93 Å for aromatic H, and constrained to ride on their parent atoms, with U_iso_(H) = xU_eq_(C, N, O), where x = 1.2 for all other H atoms.

### Crystal data

**Table:**

C_7_H_6_BrNO_2_	*F*(000) = 848
*M**_r_* = 216.04	*D*_x_ = 1.899 Mg m^−^^3^
Orthorhombic, *P**n**a*2_1_	Mo *K*α radiation, λ = 0.71073 Å
Hall symbol: P 2c -2n	Cell parameters from 3169 reflections
*a* = 24.3968 (11) Å	θ = 1.7–28.7°
*b* = 4.8388 (2) Å	µ = 5.38 mm^−^^1^
*c* = 12.8040 (5) Å	*T* = 296 K
*V* = 1511.53 (11) Å^3^	Prismatic, colorless
*Z* = 8	0.22 × 0.16 × 0.14 mm

### Data collection

**Table:**

Bruker Kappa APEXII CCD diffractometer	3908 independent reflections
Radiation source: fine-focus sealed tube	3169 reflections with *I* > 2σ(*I*)
graphite	*R*_int_ = 0.027
Detector resolution: 7.40 pixels mm^-1^	θ_max_ = 28.7°, θ_min_ = 1.7°
ω scans	*h* = −32→33
Absorption correction: multi-scan (*SADABS*; Bruker, 2005)	*k* = −6→5
*T*_min_ = 0.375, *T*_max_ = 0.469	*l* = −17→17
9922 measured reflections	

### Refinement

**Table:**

Refinement on *F*^2^	Secondary atom site location: difference Fourier map
Least-squares matrix: full	Hydrogen site location: inferred from neighbouring sites
*R*[*F*^2^ > 2σ(*F*^2^)] = 0.030	H atoms treated by a mixture of independent and constrained refinement
*wR*(*F*^2^) = 0.059	*w* = 1/[σ^2^(*F*_o_^2^) + (0.0107*P*)^2^] where *P* = (*F*_o_^2^ + 2*F*_c_^2^)/3
*S* = 1.00	(Δ/σ)_max_ < 0.001
3908 reflections	Δρ_max_ = 0.44 e Å^−^^3^
214 parameters	Δρ_min_ = −0.49 e Å^−^^3^
1 restraint	Absolute structure: Flack (1983), 1857 Friedel pairs
Primary atom site location: structure-invariant direct methods	Flack parameter: 0.012 (9)

### Special details

**Table:**

Geometry. All e.s.d.'s (except the e.s.d. in the dihedral angle between two l.s. planes) are estimated using the full covariance matrix. The cell e.s.d.'s are taken into account individually in the estimation of e.s.d.'s in distances, angles and torsion angles; correlations between e.s.d.'s in cell parameters are only used when they are defined by crystal symmetry. An approximate (isotropic) treatment of cell e.s.d.'s is used for estimating e.s.d.'s involving l.s. planes.
Refinement. Refinement of *F*^2^ against ALL reflections. The weighted *R*-factor *wR* and goodness of fit *S* are based on *F*^2^, conventional *R*-factors *R* are based on *F*, with *F* set to zero for negative *F*^2^. The threshold expression of *F*^2^ > σ(*F*^2^) is used only for calculating *R*-factors(gt) *etc*. and is not relevant to the choice of reflections for refinement. *R*-factors based on *F*^2^ are statistically about twice as large as those based on *F*, and *R*- factors based on ALL data will be even larger.

### Fractional atomic coordinates and isotropic or equivalent isotropic displacement parameters (Å^2^)

**Table:**

	*x*	*y*	*z*	*U*_iso_*/*U*_eq_	
Br1	0.320114 (15)	−0.05594 (7)	0.25848 (2)	0.03957 (10)	
O1	0.14368 (11)	0.8516 (5)	0.40595 (16)	0.0440 (6)	
H1	0.1288	0.9846	0.3781	0.053*	
O2	0.16062 (9)	0.7357 (4)	0.24087 (17)	0.0350 (5)	
N1	0.33671 (15)	0.0202 (8)	0.4953 (3)	0.0510 (10)	
H1A	0.3479 (16)	−0.127 (8)	0.455 (3)	0.061*	
H1B	0.3387 (19)	0.004 (8)	0.560 (4)	0.061*	
C1	0.21062 (12)	0.5149 (6)	0.3765 (3)	0.0302 (6)	
C2	0.23965 (12)	0.3438 (6)	0.3108 (2)	0.0288 (7)	
H2	0.2318	0.3416	0.2397	0.035*	
C3	0.28043 (13)	0.1752 (6)	0.3501 (2)	0.0275 (7)	
C4	0.29402 (14)	0.1757 (7)	0.4569 (3)	0.0339 (8)	
C5	0.26337 (14)	0.3430 (8)	0.5214 (3)	0.0428 (9)	
H5	0.2702	0.3409	0.5928	0.051*	
C6	0.22328 (15)	0.5119 (7)	0.4833 (3)	0.0416 (9)	
H6	0.2041	0.6265	0.5287	0.050*	
C7	0.16965 (14)	0.7060 (7)	0.3350 (3)	0.0312 (8)	
Br2	0.068709 (14)	−0.02802 (6)	0.87184 (3)	0.03738 (9)	
O3	−0.09440 (11)	0.8621 (5)	0.66215 (19)	0.0441 (6)	
H3	−0.1136	0.9859	0.6862	0.053*	
O4	−0.08170 (10)	0.7685 (5)	0.83152 (18)	0.0400 (6)	
N2	0.09823 (15)	−0.0184 (7)	0.6365 (3)	0.0468 (9)	
H2A	0.1139 (15)	−0.152 (7)	0.687 (3)	0.056*	
H2B	0.1093 (17)	−0.052 (8)	0.580 (4)	0.056*	
C8	−0.02918 (14)	0.5234 (6)	0.7088 (3)	0.0284 (7)	
C9	−0.00474 (13)	0.3632 (6)	0.7867 (2)	0.0281 (7)	
H9	−0.0167	0.3786	0.8554	0.034*	
C10	0.03682 (12)	0.1830 (6)	0.7625 (3)	0.0286 (6)	
C11	0.05602 (13)	0.1496 (7)	0.6603 (3)	0.0305 (7)	
C12	0.03023 (14)	0.3106 (8)	0.5824 (2)	0.0400 (8)	
H12	0.0416	0.2949	0.5134	0.048*	
C13	−0.01099 (15)	0.4883 (6)	0.6068 (3)	0.0371 (8)	
H13	−0.0275	0.5894	0.5537	0.045*	
C14	−0.07084 (13)	0.7263 (6)	0.7391 (3)	0.0315 (7)	

### Atomic displacement parameters (Å^2^)

**Table:**

	*U*^11^	*U*^22^	*U*^33^	*U*^12^	*U*^13^	*U*^23^
Br1	0.03529 (19)	0.04090 (18)	0.0425 (2)	0.00405 (15)	0.00077 (17)	−0.0027 (2)
O1	0.0561 (17)	0.0506 (15)	0.0252 (12)	0.0258 (13)	0.0033 (11)	0.0052 (11)
O2	0.0373 (13)	0.0412 (13)	0.0265 (13)	0.0111 (9)	0.0002 (10)	0.0058 (10)
N1	0.049 (2)	0.069 (2)	0.0351 (19)	0.0173 (16)	−0.0101 (17)	0.0089 (16)
C1	0.0271 (15)	0.0389 (15)	0.0246 (15)	0.0026 (13)	−0.0001 (16)	0.0062 (15)
C2	0.0250 (18)	0.0351 (18)	0.0264 (17)	−0.0012 (14)	−0.0023 (13)	0.0040 (14)
C3	0.0254 (17)	0.0300 (16)	0.027 (2)	0.0002 (13)	0.0000 (13)	0.0040 (13)
C4	0.0293 (19)	0.0360 (19)	0.036 (2)	0.0029 (15)	−0.0073 (14)	0.0071 (16)
C5	0.044 (2)	0.060 (2)	0.0246 (17)	0.0105 (19)	−0.0050 (16)	−0.0015 (17)
C6	0.039 (2)	0.051 (2)	0.034 (2)	0.0133 (16)	0.0008 (16)	0.0011 (16)
C7	0.032 (2)	0.0305 (17)	0.0310 (19)	−0.0003 (14)	0.0025 (15)	0.0055 (15)
Br2	0.03815 (19)	0.03913 (17)	0.03487 (17)	0.00381 (15)	−0.00324 (17)	0.0049 (2)
O3	0.0484 (16)	0.0458 (15)	0.0381 (14)	0.0211 (12)	−0.0097 (12)	−0.0080 (12)
O4	0.0403 (16)	0.0471 (15)	0.0326 (13)	0.0124 (12)	−0.0019 (11)	−0.0078 (11)
N2	0.053 (2)	0.054 (2)	0.0327 (18)	0.0179 (16)	0.0056 (16)	−0.0052 (16)
C8	0.0292 (17)	0.0257 (15)	0.0303 (17)	0.0030 (13)	−0.0028 (14)	−0.0054 (13)
C9	0.0309 (18)	0.0323 (16)	0.0212 (16)	−0.0029 (14)	0.0027 (12)	−0.0047 (12)
C10	0.0310 (16)	0.0269 (14)	0.0278 (16)	−0.0003 (12)	−0.0057 (15)	0.0013 (16)
C11	0.0285 (17)	0.0335 (17)	0.0296 (17)	0.0008 (14)	−0.0003 (14)	−0.0041 (15)
C12	0.044 (2)	0.055 (2)	0.0206 (17)	0.0048 (17)	0.0046 (15)	−0.0024 (15)
C13	0.046 (2)	0.0403 (17)	0.0254 (19)	0.0058 (16)	−0.0058 (15)	0.0011 (14)
C14	0.0302 (17)	0.0287 (16)	0.035 (2)	0.0012 (12)	−0.0015 (16)	−0.0019 (14)

### Geometric parameters (Å, °)

**Table:**

Br1—C3	1.888 (3)	Br2—C10	1.899 (3)
O1—C7	1.312 (4)	O3—C14	1.316 (4)
O1—H1	0.8200	O3—H3	0.8200
O2—C7	1.234 (3)	O4—C14	1.230 (3)
N1—C4	1.376 (4)	N2—C11	1.347 (5)
N1—H1A	0.92 (4)	N2—H2A	0.99 (4)
N1—H1B	0.83 (4)	N2—H2B	0.79 (4)
C1—C2	1.376 (5)	C8—C13	1.390 (5)
C1—C6	1.402 (5)	C8—C9	1.397 (4)
C1—C7	1.462 (5)	C8—C14	1.465 (4)
C2—C3	1.382 (4)	C9—C10	1.373 (4)
C2—H2	0.9300	C9—H9	0.9300
C3—C4	1.407 (4)	C10—C11	1.400 (5)
C4—C5	1.377 (5)	C11—C12	1.413 (5)
C5—C6	1.365 (5)	C12—C13	1.360 (5)
C5—H5	0.9300	C12—H12	0.9300
C6—H6	0.9300	C13—H13	0.9300
			
C7—O1—H1	109.5	C14—O3—H3	109.5
C4—N1—H1A	116 (2)	C11—N2—H2A	123 (2)
C4—N1—H1B	117 (3)	C11—N2—H2B	126 (3)
H1A—N1—H1B	118 (4)	H2A—N2—H2B	109 (4)
C2—C1—C6	118.5 (3)	C13—C8—C9	117.8 (3)
C2—C1—C7	120.7 (3)	C13—C8—C14	123.5 (3)
C6—C1—C7	120.7 (3)	C9—C8—C14	118.7 (3)
C1—C2—C3	120.2 (3)	C10—C9—C8	120.5 (3)
C1—C2—H2	119.9	C10—C9—H9	119.7
C3—C2—H2	119.9	C8—C9—H9	119.7
C2—C3—C4	121.5 (3)	C9—C10—C11	122.1 (3)
C2—C3—Br1	119.5 (2)	C9—C10—Br2	118.6 (2)
C4—C3—Br1	119.0 (2)	C11—C10—Br2	119.4 (2)
N1—C4—C5	121.3 (3)	N2—C11—C10	122.5 (3)
N1—C4—C3	121.6 (3)	N2—C11—C12	120.9 (3)
C5—C4—C3	117.1 (3)	C10—C11—C12	116.6 (3)
C6—C5—C4	121.8 (3)	C13—C12—C11	121.0 (3)
C6—C5—H5	119.1	C13—C12—H12	119.5
C4—C5—H5	119.1	C11—C12—H12	119.5
C5—C6—C1	120.8 (3)	C12—C13—C8	122.0 (3)
C5—C6—H6	119.6	C12—C13—H13	119.0
C1—C6—H6	119.6	C8—C13—H13	119.0
O2—C7—O1	121.8 (3)	O4—C14—O3	122.9 (3)
O2—C7—C1	123.4 (3)	O4—C14—C8	121.0 (3)
O1—C7—C1	114.7 (3)	O3—C14—C8	116.1 (3)
			
C6—C1—C2—C3	−0.4 (4)	C13—C8—C9—C10	1.7 (5)
C7—C1—C2—C3	176.3 (3)	C14—C8—C9—C10	−176.0 (3)
C1—C2—C3—C4	−0.9 (5)	C8—C9—C10—C11	−0.7 (5)
C1—C2—C3—Br1	−179.5 (2)	C8—C9—C10—Br2	179.7 (2)
C2—C3—C4—N1	−176.1 (3)	C9—C10—C11—N2	177.3 (3)
Br1—C3—C4—N1	2.5 (5)	Br2—C10—C11—N2	−3.1 (4)
C2—C3—C4—C5	2.7 (5)	C9—C10—C11—C12	−0.2 (5)
Br1—C3—C4—C5	−178.7 (3)	Br2—C10—C11—C12	179.4 (2)
N1—C4—C5—C6	175.6 (4)	N2—C11—C12—C13	−177.4 (3)
C3—C4—C5—C6	−3.2 (6)	C10—C11—C12—C13	0.1 (5)
C4—C5—C6—C1	2.0 (6)	C11—C12—C13—C8	1.0 (6)
C2—C1—C6—C5	−0.1 (5)	C9—C8—C13—C12	−1.8 (5)
C7—C1—C6—C5	−176.8 (3)	C14—C8—C13—C12	175.7 (3)
C2—C1—C7—O2	−3.7 (5)	C13—C8—C14—O4	−172.8 (3)
C6—C1—C7—O2	172.9 (3)	C9—C8—C14—O4	4.7 (5)
C2—C1—C7—O1	178.0 (3)	C13—C8—C14—O3	5.7 (5)
C6—C1—C7—O1	−5.3 (5)	C9—C8—C14—O3	−176.8 (3)

### Hydrogen-bond geometry (Å, °)

**Table:**

*D*—H···*A*	*D*—H	H···*A*	*D*···*A*	*D*—H···*A*
O1—H1···O4^i^	0.82	1.76	2.564 (3)	165
N1—H1A···Br1	0.92 (4)	2.63 (4)	3.081 (4)	111 (3)
N1—H1B···O2^ii^	0.83 (5)	2.57 (5)	3.313 (4)	149 (4)
N2—H2A···Br2	0.99 (4)	2.68 (4)	3.099 (4)	106 (2)
N2—H2A···Br1^ii^	0.99 (4)	2.69 (4)	3.630 (4)	158 (3)
N2—H2B···O1^iii^	0.79 (5)	2.43 (5)	3.216 (4)	179 (6)
O3—H3···O2^iv^	0.82	1.90	2.723 (3)	178
C5—H5···O2^ii^	0.93	2.59	3.407 (4)	147
C12—H12···O4^v^	0.93	2.54	3.470 (4)	174

Symmetry codes: (i) −*x*, −*y*+2, *z*−1/2; (ii) −*x*+1/2, *y*−1/2, *z*+1/2; (iii) *x*, *y*−1, *z*; (iv) −*x*, −*y*+2, *z*+1/2; (v) −*x*, −*y*+1, *z*−1/2.
